# Acute Stroke Care in the With-COVID-19 Era: Experience at a Comprehensive Stroke Center in Japan

**DOI:** 10.3389/fneur.2020.611504

**Published:** 2021-01-18

**Authors:** Junpei Koge, Masayuki Shiozawa, Kazunori Toyoda

**Affiliations:** Department of Cerebrovascular Medicine, National Cerebral and Cardiovascular Center, Suita, Japan

**Keywords:** acute ischemic stroke, COVID-19, neuroimaging, thrombolysis, mechanical thrombectomy

## Abstract

**Introduction:** The pandemic of coronavirus disease 2019 (COVID-19) has had a significant impact on stroke healthcare, including the prehospital care system and in-hospital workflow. Japan experienced the outbreak of COVID-19, and the State of Emergency was declared during April 2020 and May 2020. The aim of the present study was to clarify the effect of the COVID-19 pandemic on a comprehensive stroke center in Japan.

**Methods:** We retrospectively reviewed consecutive patients with acute ischemic stroke admitted in our institute between December 2019 and July 2020. The patients who underwent reperfusion therapy (intravenous thrombolysis and/or mechanical thrombectomy) were divided into the pre-COVID-19 period (December 2019 to March 2020) and the With-COVID-19 period (April 2020 to July 2020). Study outcomes were the number of stroke admissions in our institute, workflow time metrics, the frequency of modified Rankin Scale score 0–2 at discharge, and brain imaging modalities before reperfusion therapy in patients who underwent reperfusion therapy.

**Results:** In our institute, the number of stroke admissions decreased during the State of Emergency and then increased after the lifting of the State of Emergency. Among patients who underwent reperfusion therapy (median age, 77 years; female 27%; median baseline National Institutes of Health Stroke Scale score, 10), times from hospital arrival to imaging [25 (21–33) min vs. 30 (25–38) min, *P* = 0.03] and to thrombolysis [38 (31–52) min vs. 51 (37–64) min, *P* = 0.03] were prolonged compared with the pre-COVID-19 period. There was no significant difference in the frequency of modified Rankin Scale score 0–2 at discharge between the two periods (32 vs. 45%, *P* = 0.21). The proportion of computed tomography vs. magnetic resonance imaging as an emergency brain imaging tool before reperfusion therapy changed, with computed tomography having become predominant in the With-COVID-19 period.

**Conclusions:** In our institute, the number of stroke admissions, workflow time metrics, and imaging modalities for reperfusion therapy were affected by the COVID-19 pandemic.

## Introduction

The emergence of a novel coronavirus, severe acute respiratory syndrome coronavirus 2 (SARS-CoV-2), in Wuhan in December 2019 evolved into a pandemic that was declared on March 11, 2020 (1). As of September 9, 2020, a total of 27,477,869 patients had been reported, with 896,173 deaths worldwide (2). Although the most common presentation of patients with coronavirus disease 2019 (COVID-19) is symptoms due to respiratory disease, the clinical presentation of patients with COVID-19 varies considerably, ranging from asymptomatic infection to multiple organ failure. Recently, reports regarding the neurological manifestations of COVID-19 have been increasing: 8.0% of patients treated for COVID-19 presented with a preexisting neurologic illness (3), and it is estimated that 4.9% of COVID-19 patients have acute stroke (4). Healthcare providers engaged in neurological emergency care are inevitably at risk of COVID-19 exposure. Consequently, the COVID-19 global pandemic has a great impact on every aspect of emergency stroke healthcare, including the prehospital care system and the in-hospital workflow (5–7). In light of the in-hospital workflow, modified in-hospital stroke protocols designed to protect against COVID-19 transmission have been proposed (Protected Code Stroke) (8). Stroke team members are faced with the novel and significant challenge of providing high-quality emergency treatment while continuing their utmost effort to minimize infectious exposure. From the perspective of the prehospital care system, significant delays in stroke onset to hospital arrival time (9) and reduction of stroke admissions have been reported (10, 11).

**Figure 1:**
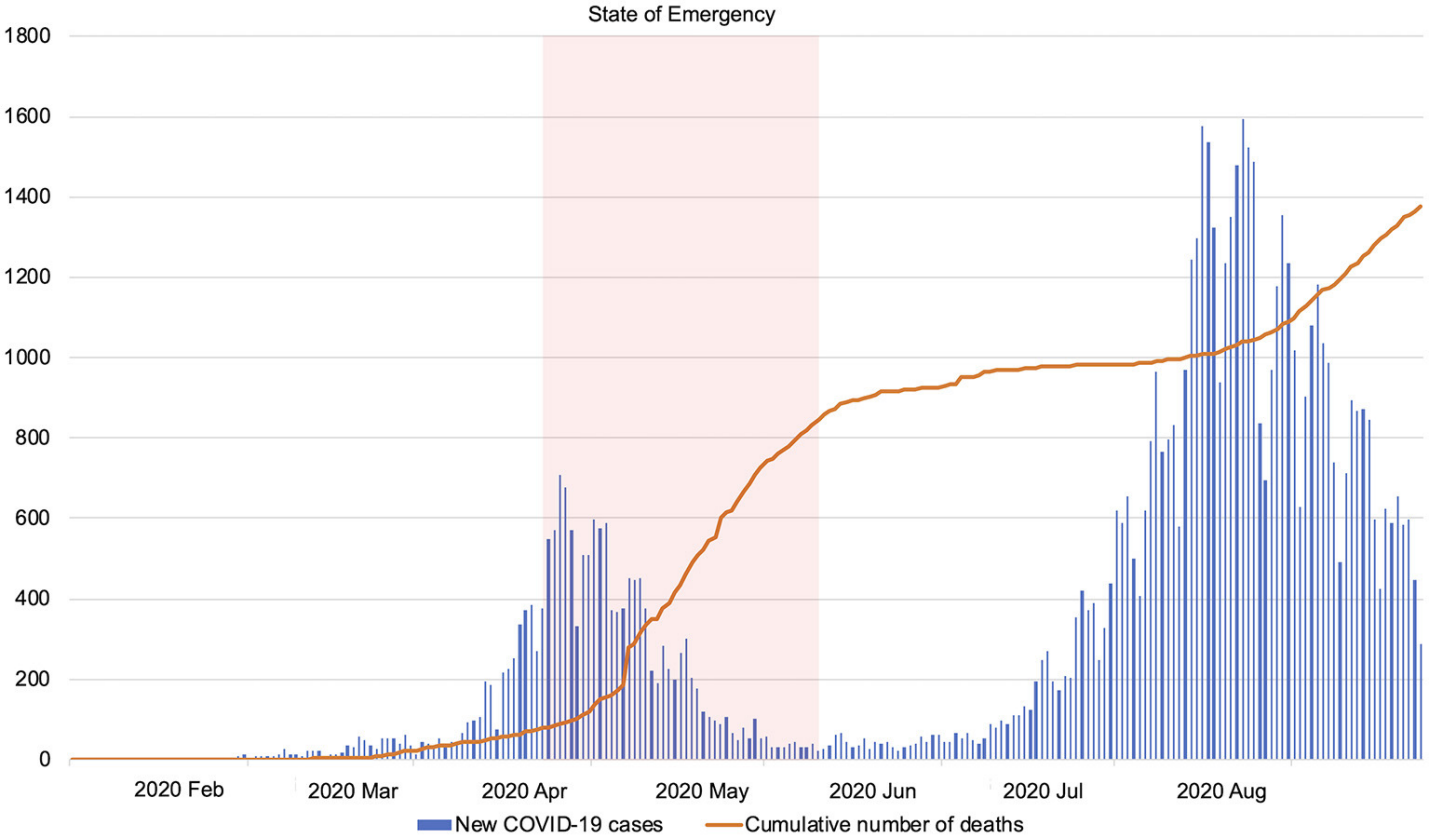
The number of new COVID-19 cases and cumulative number of deaths in Japan (January 6, 2020–September 7, 2020). The State of Emergency was declared on April 7, 2020, and lifted on May 25, 2020. Edited based on the openly available data from the Ministry of Health, Labor and Welfare, Japan (https://www.mhlw.go.jp/stf/covid-19/open-data.html, in Japanese). COVID-19, coronavirus disease 2019.

Japan recorded its first COVID-19 patient on January 16, 2020, and experienced rapid spread of infection, mainly in urban areas. The State of Emergency was declared on April 7, 2020, for urban areas, and the declaration was extended to the rest of the country on April 16, 2020. After the declaration, the curve of infection spread flattened slowly. The State of Emergency was lifted on May 25, 2020. The number of COVID-19-positive cases increased again rapidly after the lifting of the declaration. As of September 9, 2020, the numbers of domestic infections and deaths reached 71,873 and 1,376, respectively (12). The mortality rate has remained relatively low, at 1.9%. The new COVID-19 cases and the cumulative number of COVID-19 deaths in Japan are shown in Figure 1.

On April 28, 2020, the Japan Stroke Society, an academic organization, published the Japan Stroke Society-Protected Code Stroke (13). The Japan Stroke Society-Protected Code Stroke was designated with priority on protecting stroke team members from COVID-19 infection exposure based on Protected Code Stroke (8). The Japan Stroke Society-Protected Code Stroke proposed the following main points: [1] to regard all stroke patients presenting to the emergency department as possibly infectious; [2] the appropriate use of personal protective equipment (PPE) and placement of a surgical mask on non-intubated patients; [3] preferential use of computed tomography (CT) as a neuroimaging modality with a chest CT scan to screen for findings suggestive of COVID-19 infection; and [4] to keep to a minimum medical staff involved in acute stroke care at the emergency department. Magnetic resonance imaging (MRI) was frequently used as the major diagnostic tool for acute stroke in Japan most recently (14), but the proportion of CT vs. MRI as an emergency brain imaging tool for acute stroke might change in many institutions, since CT has now become predominant.

As of the time of writing (September 2020), the outbreak in Japan has lasted about 6 months, including the second upward-sloping curve of spreading COVID-19 infection. In this paper, we aimed to investigate the impact of the COVID-19 pandemic on the healthcare system of acute stroke in a comprehensive stroke center in Japan. This paper focuses on current experience and highlighting the problems in providing optimized stroke healthcare for acute stroke patients in the With-COVID-19 era.

## Methods

### Study Population

This was a single-center, observational cohort study performed at a comprehensive stroke center in Japan. We retrospectively reviewed consecutive patients with acute ischemic stroke admitted in our institute between December 2019 and July 2020. The patients who underwent reperfusion therapy (intravenous thrombolysis and/or mechanical thrombectomy) were stratified into the pre-COVID-19 period (December 2019 to March 2020) and the With-COVID-19 period (April 2020 to July 2020). An ethics committee approved a series of clinical studies including this study using the National Cerebral and Cardiovascular Center (NCVC) Stroke Registry (M23-073-7).

### Acute Stroke Care in Suita City and the National Cerebral Cardiovascular Center

In Suita city (the north suburban city of metropolitan Osaka, total population of 375,000 people), acute stroke care is provided through a network of four acute hospitals. The emergency medical service (EMS) provides the urgent patient transport system with priority given to patients with suspected acute stroke brought to the nearest hospital with the appropriate diagnostic and therapeutic capacity. The NCVC is an urban comprehensive stroke center with 550 beds in Suita. The stroke service at the NCVC has a Stroke Care Unit with 18 beds managed by a multidisciplinary team of vascular neurologists, neurosurgeons, and neurointerventionalists. More than 1,000 patients with acute stroke/transient ischemic attack are hospitalized in our center every year, and roughly 150 acute reperfusion treatments including 70 mechanical thrombectomies are performed. The number of hospitalized patients in 2019 was 690 for ischemic stroke, 204 for intracranial hemorrhage, 55 for subarachnoid hemorrhage, and 70 for transient ischemic attack.

### Workflow in the National Cerebral Cardiovascular Center During the COVID-19 Pandemic

At the NCVC, all stroke inpatients have undergone polymerase chain reaction (PCR) testing for COVID-19 on the morning after admission since May 1, 2020. Patients before confirmation of a negative COVID-19 PCR have been admitted to an isolated bed in the Stroke Care Unit and transferred to the clean Stroke Care Unit after confirmation of a negative COVID-19 PCR. At the time of writing (September 2020), no stroke patient with COVID-19 has been admitted to the NCVC. The 24/7 multimodal brain imaging, including MRI and CT angiography, and mechanical thrombectomy (MT) are available. The novel institutional stroke protocol for the COVID-19 pandemic was developed and implemented in April 2020. Patients with fever above 37.5°C or respiratory symptoms and suspected of COVID-19 infection by physicians were deemed as suspected COVID-19 cases. When patients were admitted in the emergency department, all stroke team members wore PPE including an N-95 mask, face shield, gown, and gloves and placed a surgical mask on non-intubated patients regardless of suspicion of COVID-19 infection. All patients underwent portable chest X-ray in the emergency department before brain imaging. Head CT and CT angiography have been prioritized for initial brain imaging over MRI. If any abnormal findings were recognized on portable chest X-ray, head CT was selected for a neuroimaging modality and concurrent chest CT was performed for further screening of COVID-19 infection. When performing MRI for patients who are not confirmed negative for COVID-19 or suspected COVID-19 cases, cleaning and ventilation for 15 min or 2 h after the scan for complete disinfection have been performed, respectively. When performing brain imaging, the roles of the physician in charge of transportation and the physician who interprets images in the control room are separated to prevent infection exposure in the control room. Patients who are not confirmed negative for COVID-19 wear surgical masks during transportation and neuroimaging. Limiting other traffic through the healthcare facility during transportation of positive/suspected COVID-19 cases is recommended. MT for patients who are not confirmed negative for COVID-19 by PCR has been performed by the minimum number (≈5) of staff wearing full PPE (N-95 mask, surgical mask, face shield, cap, gown, gloves) to limit provider exposure and the amount of protective gear used. Cleaning and ventilation of the angiography suite for 2 h after procedures for COVID-19-positive or suspected COVID-19 cases have been performed. Swallowing assessment in patients before confirmation of negative COVID-19 PCR has mainly been performed by a repetitive saliva swallowing test under the standard PPE (surgical mask, face shield, gloves). The performance of carotid ultrasound and transesophageal echocardiography before confirmation of negative COVID-19 PCR has been limited.

### Study Outcomes

The primary outcomes of the present study were [1] the number of stroke admissions and [2] reperfusion therapies in the NCVC. Other outcome measures were [1] workflow time metrics, such as from stroke onset to hospital arrival, hospital arrival to brain imaging, intravenous thrombolysis, or groin puncture, and stroke onset to intravenous thrombolysis or groin puncture; [2] modified Rankin Scale score 0–2 at discharge; and [3] brain imaging modalities before reperfusion therapy.

### Statistical Analyses

Categorical variables were presented as frequencies and percentages and compared with the Fisher's exact test. Continuous variables were presented as median and interquartile range and were compared with the Wilcoxon rank-sum test. All reported *P*-values are for a two-sided test, and *P* < 0.05 was considered significant. Statistical analyses were performed using JMP 14.2.0 software (SAS Institute, Cary, NC, USA).

## Results

### Emergency Medical Service Transfers in Suita City, Stroke Admissions, and Reperfusion Therapies in the National Cerebral Cardiovascular Center

**Figure 2:**
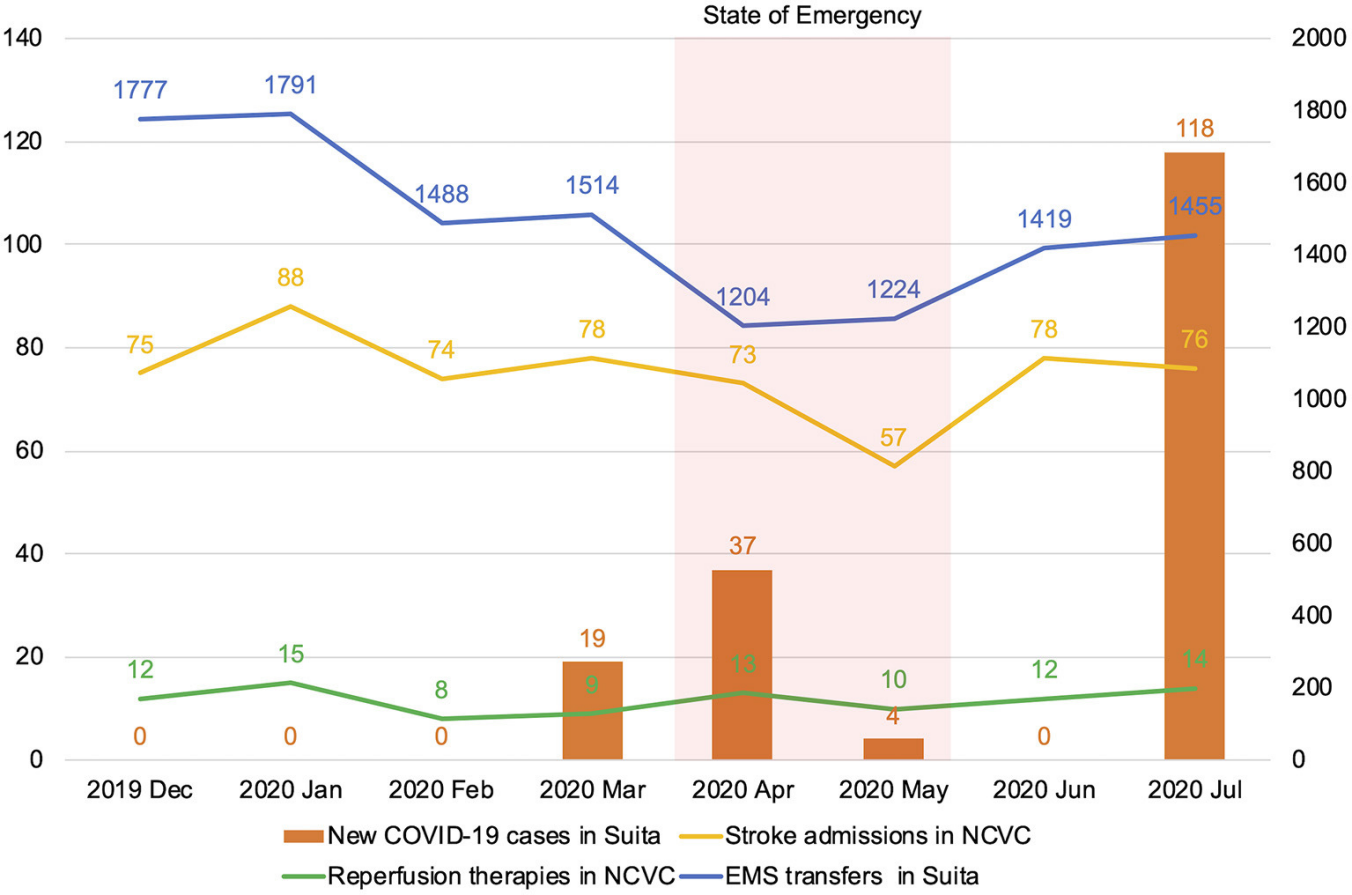
Monthly volume of emergency medical service transfers and new coronavirus 2019 (COVID-19) cases in Suita city and stroke admissions and the number of reperfusion therapy cases in the National Cerebral and Cardiovascular Center. COVID-19, coronavirus disease 2019; NCVC, National Cerebral and Cardiovascular Center; EMS, emergency medical service.

In Osaka prefecture with a population of 8,822,000 residents, the total numbers of COVID-19 infections and deaths were 9,169 and 168, respectively, as of September 9, 2020 (15). The number of EMS transfers and new COVID-19 patients in Suita city decreased gradually after the declaration of the State of Emergency. The number of stroke admissions in the NCVC also decreased during the State of Emergency. The number of patients receiving reperfusion therapies in our institute remained unchanged. After the lifting of the State of Emergency, EMS transfers in Suita city and stroke admissions in the NCVC increased. The number of COVID-19 infections has increased 1 month after the lifting of the State of Emergency (the second wave of COVID-19). However, EMS transfers in Suita city, stroke admissions, and the number of patients who underwent reperfusion therapy in the NCVC did not decrease (Figure 2).

### Workflow Time Metrics and Outcomes in Stroke Patients Who Underwent Reperfusion Therapies

The clinical characteristics and time metrics of acute stroke patients who underwent reperfusion therapy are shown in Table 1. There were no significant differences in the baseline characteristics, stroke subtypes, and treatment between the pre-COVID-19 and With-COVID-19 periods. The median time from stroke onset to hospital arrival was ≈30 min longer in the With-COVID-19 period compared with pre-COVID-19 period, though the differences were not significant. The median times from hospital arrival to brain imaging and from hospital arrival to thrombolysis were significantly longer than in the pre-COVID-19 period. There were insignificant increases of ≈10 min in median hospital arrival to groin puncture time in the With-COVID-19 period. There was no significant difference in the frequency of modified Rankin Scale score 0–2 at discharge between the two periods.

**Table 1:** In-hospital workflow metrics in the periods before and during the With-COVID-19 pandemic in the National Cerebral and Cardiovascular Center.

	**Pre-COVID-19 period**	**With-COVID-19 period**	***P*-value**
	**December 2019 to March 2020 (*N* = 44)**	**April 2020 to July 2020 (*N* = 49)**	
Age, year; median (IQR)	79 (65–86)	75 (65–86)	0.20
Female, n (%)	15 (34)	10 (20)	0.16
Premorbid mRS score, median (IQR)	0 (0–1)	0 (0–1)	0.55
Baseline NIHSS score, median (IQR)	11 (6–20)	10 (5–19)	0.56
Large artery atherosclerosis, n (%)	9 (20)	7 (14)	0.58
Cardioembolism, n (%)	23 (52)	26 (53)	1.00
Small vessel disease, (%)	1 (2)	0 (0)	0.47
Other cause, n (%)	9 (20)	11 (22)	1.00
Undetermined cause, n (%)	2 (5)	5 (10)	0.44
IVT only, n (%)	22 (50)	30 (61)	0.30
Bridging IVT with MT, n (%)	12 (27)	11 (22)	0.64
MT only, n (%)	10 (23)	8 (16)	0.60
Stroke onset to hospital arrival time, min, median (IQR)	71 (52–182)	109 (49–182)	0.54
Hospital arrival to brain imaging time, min, median (IQR)	25 (21–33)	30 (25–38)	0.03
Hospital arrival to thrombolysis time^*^, min, median (IQR)	38 (31–52)	51 (37–64)	0.03
Stroke onset to thrombolysis time^*^, min, median (IQR)	117 (89–175)	150 (106–210)	0.11
Hospital arrival to groin puncture time^†^, min, median (IQR)	70 (53–90)	82 (67–101)	0.16
Stroke onset to groin puncture time^†^, min, median (IQR)	186 (109–450)	210 (153–291)	0.79
mRS score 0–2 at discharge, n (%)	14 (32)	22 (45)	0.21

*Numbers are n (%) or median (IQR) values as appropriate. Intergroup comparisons were performed using the Wilcoxon rank-sum test*.

* *Only patients who received intravenous thrombolysis are included*.

† *Only patients who received mechanical thrombectomy are included*.

*IQR, interquartile range; IVT, intravenous thrombolysis; MT, mechanical thrombectomy; NIHSS, National Institutes of Health Stroke Scale; mRS, modified Rankin scale*.

### Brain Imaging Modalities Before Reperfusion Therapies

**Figure 3:**
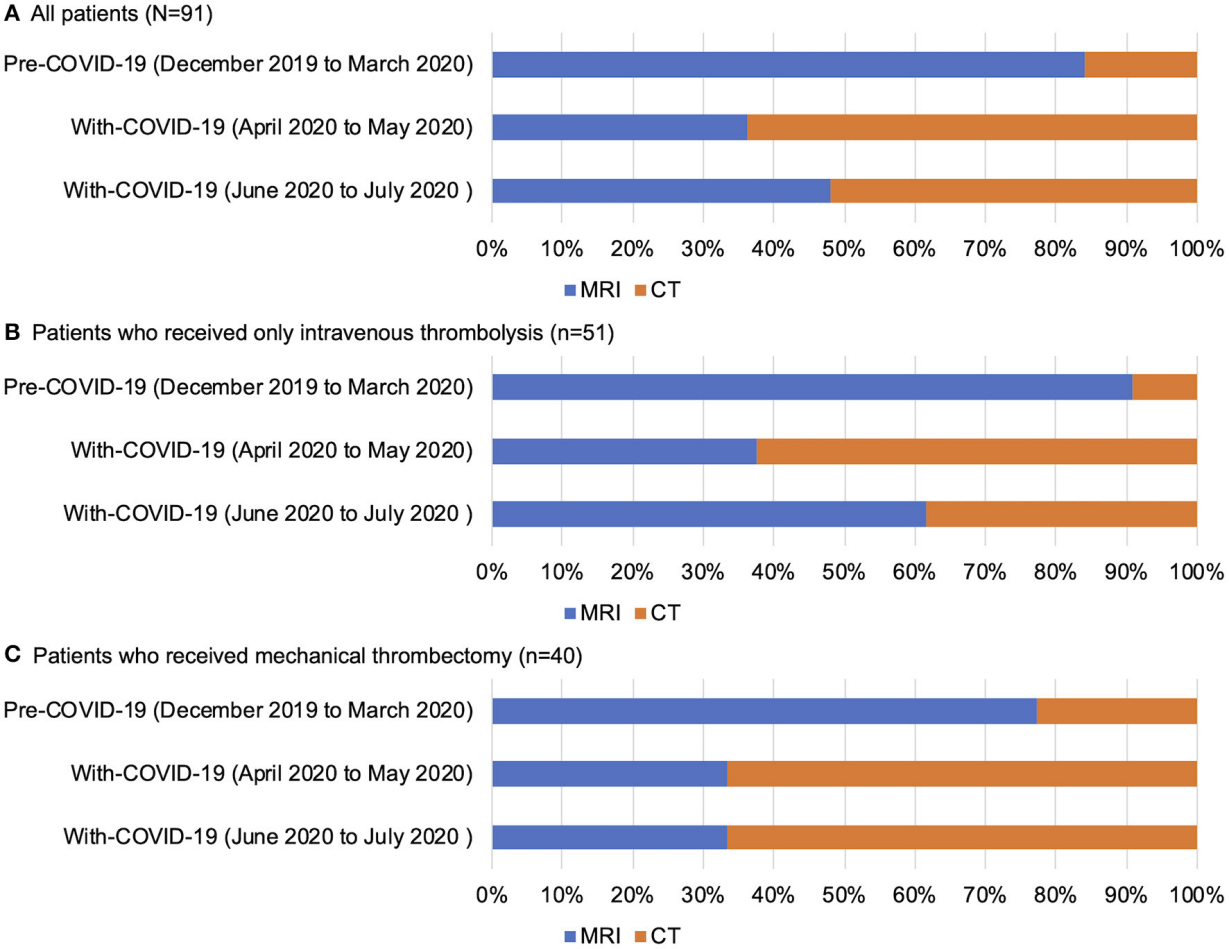
Selection of brain imaging modalities before reperfusion therapy in all patients **(A)**, patients who received only intravenous thrombolysis **(B)**, and patients who received mechanical thrombectomy **(C)** in the National Cerebral and Cardiovascular Center. COVID, coronavirus disease.

Before the COVID-19 pandemic, brain MRI was mainly used to evaluate the indication for reperfusion therapy according to the institutional policy. During the State of Emergency in the With-COVID-19 period, the proportion of patients undergoing CT increased due to the change of the institutional protocol. After the lifting of the State of Emergency in the With-COVID-19 period, the proportion of patients undergoing MRI increased especially in patients who only received intravenous thrombolysis. CT perfusion was frequently used for patients who received MT (Figure 3).

### A Representative Acute Stroke Case With Suspected COVID-19 Infection

**Figure 4:**
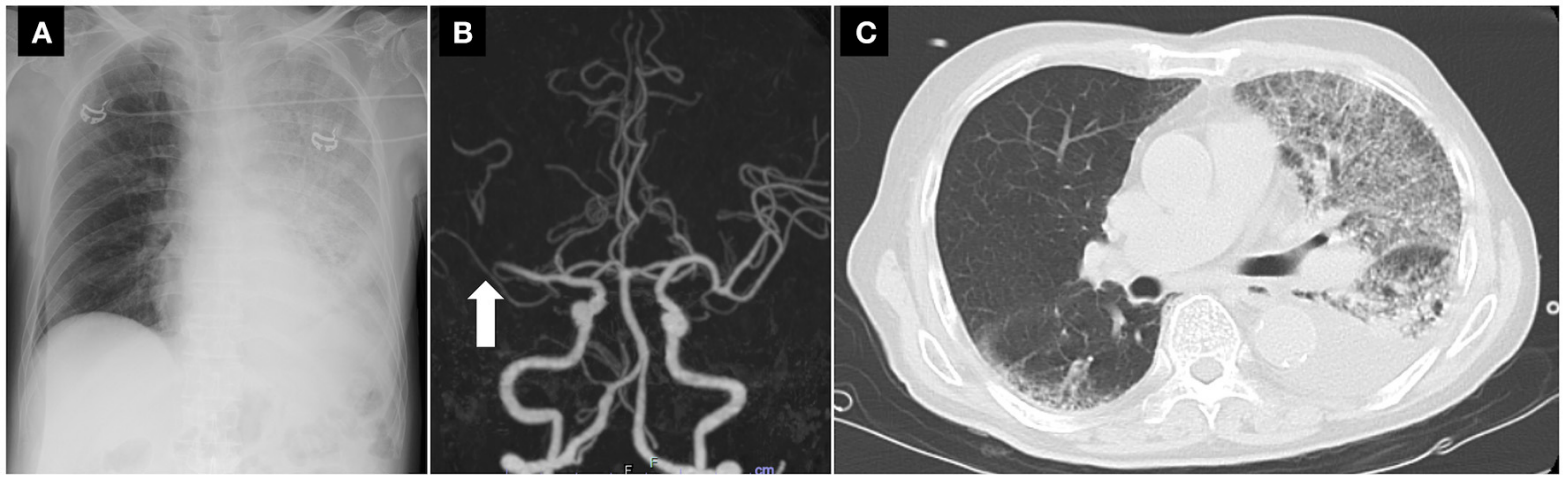
A portable X-ray, CT angiography, and chest CT findings of the stroke patient with suspected COVID-19. **(A)** A portable chest X-ray showing extensive consolidation in the left lung. **(B)** CT angiography showing an occlusion of the distal portion of the right middle cerebral artery (*arrow*). **(C)** Chest CT showing extensive consolidation in the left lung and pleural effusion in the left side. CT, computed tomography.

A patient in his mid-80's presented as an emergency to our institute with acute onset of left hemiparesis. He had had a fever and cough 2 weeks before admission. He was transferred to our hospital 59 min after symptom onset. His temperature was 37.5°C. Neurologically, he had a disturbance of consciousness, conjugate gaze preference toward the right side, and left hemiparesis, with a baseline National Institutes of Health Stroke Scale (NIHSS) score of 21. Oxygen saturation was 88% on room air. He was treated as a suspected COVID-19 case due to his presentation. All stroke team members wore PPE including an N-95 mask, face shield, gown, and gloves and performed an examination. A portable chest X-ray showed extensive consolidation in the left lung (Figure 4A). According to the institutional protocol, he underwent CT perfusion to determine eligibility for reperfusion therapy and chest CT was performed simultaneously. The time from hospital arrival to brain imaging was 22 min. Non-contrast CT showed no early ischemic changes. CT angiography showed an occlusion of the distal segment of the horizontal portion of the right middle cerebral artery (Figure 4B). CT perfusion showed the target mismatch in the area of the right middle cerebral artery. On chest CT, there was extensive consolidation in the left lung and pleural effusion in the left side (Figure 4C). He underwent thrombolysis and MT under local anesthesia. The times from hospital arrival to thrombolysis and to puncture were 34 and 83 min, respectively. While the patient was transported from the emergency department to the angiography suite, use of the flow line by other staff and patients was prohibited. Entry to the angiography suite was restricted to a minimum number of staff equipped with PPE. One pass with combined use of the stent retriever and aspiration catheter for the right middle cerebral artery occlusion retrieved white thrombi and achieved complete reperfusion. The groin puncture to reperfusion time was 25 min. On postoperative day 1, his neurological symptoms improved remarkably, and the NIHSS score was 2. Although his PCR testing for COVID-19 was negative, the consolidation on chest CT deteriorated. On postoperative day 5, he was transferred to another hospital for treatment of respiratory disease suspected to be organizing pneumonia.

## Discussion

The major findings of the present study were: [1] the number of stroke admissions in our institute decreased during the State of Emergency and increased after the lifting of the State of Emergency. The number of patients receiving reperfusion therapy remained stable; [2] times from hospital arrival to brain imaging and to thrombolysis were prolonged in the With-COVID-19 period compared with those in the pre-COVID-19 period, though clinical outcomes remained similar; [3] CT as an emergency brain imaging tool before reperfusion therapy became predominant in the With-COVID-19 period.

Acute stroke has been reported to occur in 2.8% of COVID-19-positive patients in Wuhan (16), and young-onset stroke with COVID-19 infection was reported to be marked from a hospital in New York City in early spring, 2020 (17). As a possible reason, infection has been thought to be associated with subsequent ischemic stroke (18). However, stroke admissions decreased in more recent reports during the COVID-19 pandemic in real-world settings (10, 11). A decrease was also observed in coronary artery disease admissions during the COVID-19 pandemic (19). The number of stroke admissions also decreased in our institute. Despite the decrease in the number of EMS transfers in our region, the number receiving reperfusion therapy in our institute did not decrease. A reason for this paradoxical finding might be that only patients with mild symptoms would hesitate to call EMS due to fear of COVID-19 contamination, but moderate to severely ill patients would not.

Increases in EMS transfers and stroke admissions after the lifting of the State of Emergency declaration implicate that social restrictions rather than the COVID-19 pandemic may be associated with the decreased number of stroke admissions. Medication non-adherence due to refraining from attending clinics might lead to the increased number of stroke admissions after lifting the State of Emergency. Poor management of risk factors due to lack of exercise during the State of Emergency might also contribute to the increased risk of stroke.

Head CT as emergency brain imaging modality may have advantages during the COVID-19 pandemic, including short imaging time and screening for COVID-19 infection using concurrent chest CT scan. MRI has disadvantages, including the difficulty of ventilation and disinfection of equipment, uncertainty of body search for magnetic materials of staff with heavy PPE, and long study time. The American College of Radiology recommends to minimize the use of MRI except where absolutely necessary (20). Use of MRI for patients before confirmation of negative COVID-19 PCR had been limited during the first stage of the COVID-19 pandemic in our institute. However, in recent months, the number of cases undergoing MRI has been increasing. Although MRI-based patient selection has the drawback of time-consuming disinfection procedures after the scan, it has the advantage of the identification of diffusion-weighted imaging fluid-attenuated inversion recovery mismatch, early-onset lesions, and small lesions. Recently, we have selected the brain imaging modality according to the risk of COVID-19 infection (fever, exposures to anyone with known or suspected COVID-19 within the past 14 days, respiratory symptoms, abnormal findings on chest X-ray), stroke onset to hospital arrival time, and the severity of stroke. CT perfusion has been prioritized to judge the patient's eligibility for MT because its capability for selecting patients for MT is equal to that of MRI (21).

In our institute, the times from hospital arrival to brain imaging and to thrombolysis were longer in the With-COVID-19 period. Delays from hospital arrival to brain imaging might be explained by COVID-19 screening with chest X-ray in the emergency department before transport to imaging, infection prevention precautions in the emergency department, and changes to an unfamiliar stroke protocol. The delay in the hospital arrival to groin puncture time was small, but it was due to time saving by the preferred use of CT for imaging. Although the workflow that emphasizes infection control has been proposed (22), it is unclear whether the strategy can achieve the same time metrics before the COVID-19 pandemic. A multicenter, observational study in the COVID-19 era has reported a significant increase in the mean stroke onset-to-groin puncture time (23). However, recent studies from a tertiary level center have reported no clear delays in in-hospital time metrics, including door to imaging time or thrombolysis time (5, 9, 10). These studies have shown that tertiary level centers with abundant resources including multidisciplinary teams may be able to maintain in-hospital workflow metrics during the pandemic. Since the organized in-hospital strategy that balances infection control and time saving may require a multidisciplinary team approach, the strategy may be better suited for tertiary level centers. The imaging protocol including chest CT in addition to head CT may provide rapid screening for COVID-19 in the time-sensitive setting (24). In the With-COVID-19 era, a new in-hospital workflow that can reduce treatment times, while continuing all possible infection control measures, is warranted.

## Conclusion

The stroke health care system in our region in Japan has been significantly affected by the COVID-19 pandemic despite the relatively low incidence of COVID-19. The State of Emergency seemed to be associated with a decreased number of EMS transfers and stroke admissions. In our institute, the institutional protocol for acute stroke patients was significantly modified, and some in-hospital time metrics after the COVID-19 pandemic were prolonged compared with those before the COVID-19 pandemic. The optimal in-hospital workflow considering the need to mitigate in-hospital COVID-19 transmission and a reduction in workflow time metrics has been sought in the With-COVID-19 era.

## Author Contributions

JK analyzed the data and wrote the manuscript. MS collected the data. KT supervised the manuscript. All authors contributed to the article and approved the final version of the manuscript.

## Conflict of Interest

KT received lecture honoraria from Nippon Boehringer Ingelheim, Bayer, Daiichi-Sankyo, and Bristol-Myers Squibb, outside the submitted work. The remaining authors declare that the research was conducted in the absence of any commercial or financial relationships that could be directly construed as a potential conflict of interest.
